# Long contiguous stretches of homozygosity spanning shortly the imprinted loci are associated with intellectual disability, autism and/or epilepsy

**DOI:** 10.1186/s13039-015-0182-z

**Published:** 2015-10-15

**Authors:** Ivan Y. Iourov, Svetlana G. Vorsanova, Sergei A. Korostelev, Maria A. Zelenova, Yuri B. Yurov

**Affiliations:** Mental Health Research Center, 117152 Moscow, Russia; Separated Structural Unit “Clinical Research Institute of Pediatrics”, Russian National Research Medical University named after N.I. Pirogov, Ministry of Health of Russian Federation, 125412 Moscow, Russia; Department of Medical Genetics, Russian Medical Academy of Postgraduate Education, 123995 Moscow, Russia; Research Centre for Medical Genetics, 115478 Moscow, Russia

**Keywords:** Long continuous stretches of homozygosity, Intellectual disability, Congenital anomalies, Autism, Epilepsy, Epigenetics, Bioinformatics

## Abstract

**Background:**

Long contiguous stretches of homozygosity (LCSH) (regions/runs of homozygosity) are repeatedly detected by single-nucleotide polymorphism (SNP) chromosomal microarrays. Providing important clues regarding parental relatedness (consanguinity), uniparental disomy, chromosomal recombination or rearrangements, LCSH are rarely considered as a possible epigenetic cause of neurodevelopmental disorders. Additionally, despite being relevant to imprinting, LCSH at imprinted loci have not been truly addressed in terms of pathogenicity. In this study, we examined LCSH in children with unexplained intellectual disability, autism, congenital malformations and/or epilepsy focusing on chromosomal regions which harbor imprinted disease genes.

**Results:**

Out of 267 cases, 14 (5.2 %) were found to have LCSH at imprinted loci associated with a clinical outcome. There were 5 cases of LCSH at 15p11.2, 4 cases of LCSH at 7q31.2, 3 cases of LCSH at 11p15.5, and 2 cases of LCSH at 7q21.3. Apart from a case of LCSH at 7q31.33q32.3 (~4 Mb in size), all causative LCSH were 1–1.5 Mb in size. Clinically, these cases were characterized by a weak resemblance to corresponding imprinting diseases (i.e., Silver-Russell, Beckwith-Wiedemann, and Prader-Willi/Angelman syndromes), exhibiting distinctive intellectual disability, autistic behavior, developmental delay, seizures and/or facial dysmorphisms. Parental consanguinity was detected in 8 cases (3 %), and these cases did not exhibit LCSH at imprinted loci.

**Conclusions:**

This study demonstrates that shorter LCSH at chromosomes 7q21.3, 7q31.2, 11p15.5, and 15p11.2 occur with a frequency of about 5 % in the children with intellectual disability, autism, congenital malformations and/or epilepsy. Consequently, this type of epigenetic mutations appears to be the most common one among children with neurodevelopmental diseases. Finally, since LCSH less than 2.5–10 Mb in size are generally ignored in diagnostic SNP microarray studies, one can conclude that an important epigenetic cause of intellectual disability, autism or epilepsy is actually overlooked.

## Background

The genetic causes of neurodevelopmental disorders include almost all types of genomic variations (mainly, chromosomal rearrangements (microscopic and submicroscopic), copy number variations (CNV) and single gene mutations) [1–7]. Additionally, epigenetic alterations due to genomic variations affecting genes involved in epigenomic regulation and uniparental disomy resulting from chromosomal or segmental homozygosity (HMZ) are shown to contribute to the etiology of neurodevelopmental diseases [8–11]. However, epigenomic variations and instability have been significantly less investigated in terms of the causative role for these diseases than deserved [10, 11]. Moreover, genome- and epigenome-based analysis of brain cells suggests that epigenetic changes are likely an underappreciated source of neuronal diversity and neurodevelopmental diseases [12]. Accordingly, one can hypothesize that overlooked epigenomic variations might be involved in the pathogenesis of neurological and psychiatric diseases.

Probably the commonest type of epigenomic variations in humans is long contiguous stretches of homozygosity (LCSH) (also known as regions/runs of homozygosity and losses of heterozygosity) defined as CNV neutral chromosomal segments featured by allelic HMZ [13–15]. LCSH over 1 Mb are always observed during genome-wide analyses by single-nucleotide polymorphism (SNP) chromosomal microarrays [16–19]. The presence of LCSH can be indicative for parental consanguinity, uniparental disomy, or HMZ for single gene recessive mutations [19–21]. Furthermore, LCHS are helpful for uncovering the genetic basis for complex traits [22] and locus-specific deleterious genomic variation [23]. Taking into account the epigenetic contribution to brain development and plasticity as well as genetic-environmental interactions in neuropsychiatric diseases, epigenomic variability was suggested to be a mechanism for neurodevelopmental disorders [24, 25]. Recently, several studies have addressed LCSH in brain diseases. However, these yielded conflicting results [26–28]. Actually, the only more-or-less confirmed association between LCSH occurrence and neurodevelopmental diseases (intellectual disability (ID) and autism) is related to excess of LCSH encompassing recessive disease genes [19, 29]. Surprisingly, imprinted gene loci were not considered as a target for studying LCSH in neurodevelopmental disorders. Since classical imprinting syndromes, i. e. Angelman syndrome (AS), Beckwith-Wiedemann syndrome (BWS), Prader-Willi syndrome (PWS) and Silver-Russell syndrome (SRS), are associated with ID, autistic behavior, developmental delay, and seizures [30, 31], we hypothesize that LCSH at these disease loci may result in a similar neurological or behavioral phenotype.

In this study, LCSH were evaluated in a cohort of children with idiopathic intellectual disability, autism, congenital malformations and/or epilepsy by SNP chromosomal microarray with a resolution of HMZ stretch detection reaching a minimum of 1 Mb in size. An original bioinformatics approach to the prioritization of genes and genome/epigenome variations was used to assess pathogenic value of CNV and LCSH.

## Results

The presence of LCSH was observed in all cases studied. Causative chromosome abnormalities, CNV and intragenic (exonic) CNV detected by SNP microarray technique were excluded from further analysis. Apart from individuals, who were the descendants of close consanguinity marriages, the amount of LCSH per patient varied between 63 and 132. Eight individuals (3 %) were descendants of close consanguinity marriages. Parental consanguinity was determined according to a methodology of a previous study by Fan et al. [32] and genealogic analysis. In these patients, LCSH have not spanned the loci of imprinted genes strongly associated with recognizable syndromes. A patient, who is a descendant of consanguinity mating, exhibited LCSH at 7p12, containing an imprinted gene *GRB10*. Since the involvement of *GRB10* in SRS is questionable [33], we have excluded this case from further analysis.

**Fig. 1:**
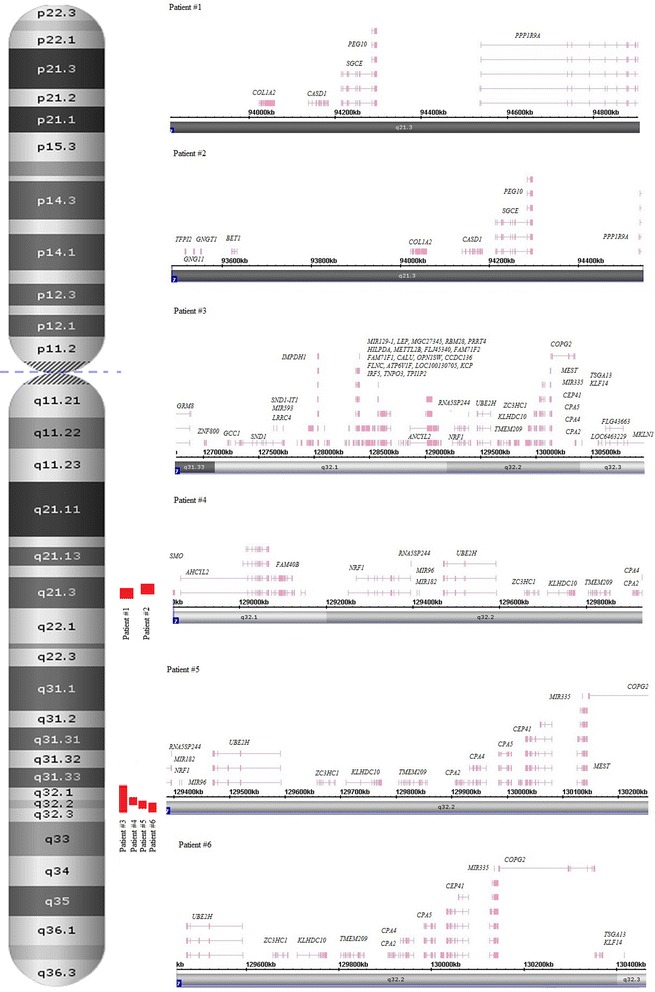
Schematic depiction of chromosomal and genomic regions affected by LCHS at 7q21.3 (2 patients) and 7q31.2 (4 patients) using Affymetrix Chromosome Analysis Suite software screenshots

**Fig. 2:**
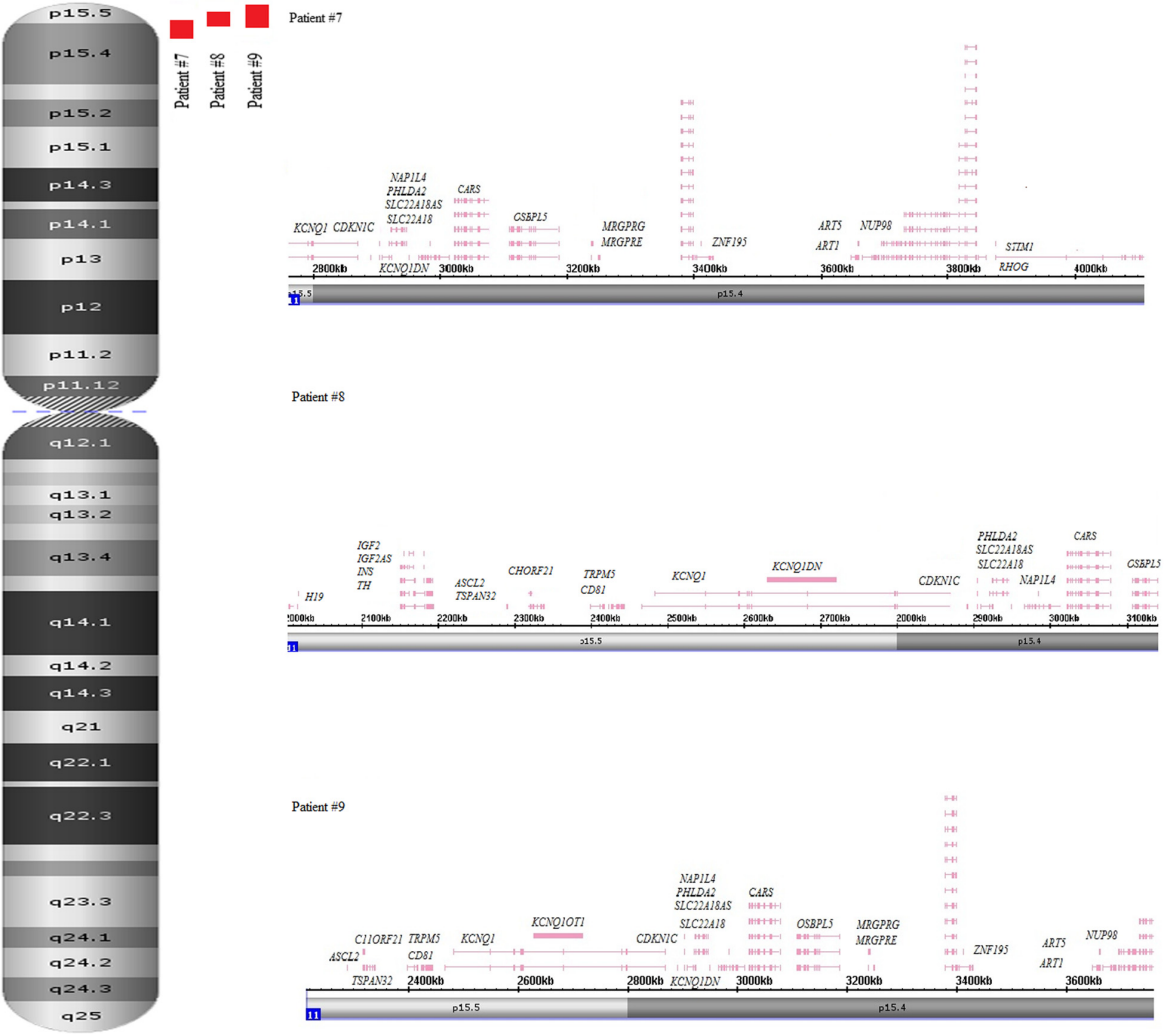
Schematic depiction of chromosomal and genomic regions affected by LCHS at 11p15.5 (11p15.5 and 11p15.4) (3 patients) using Affymetrix Chromosome Analysis Suite software screenshots

**Fig. 3:**
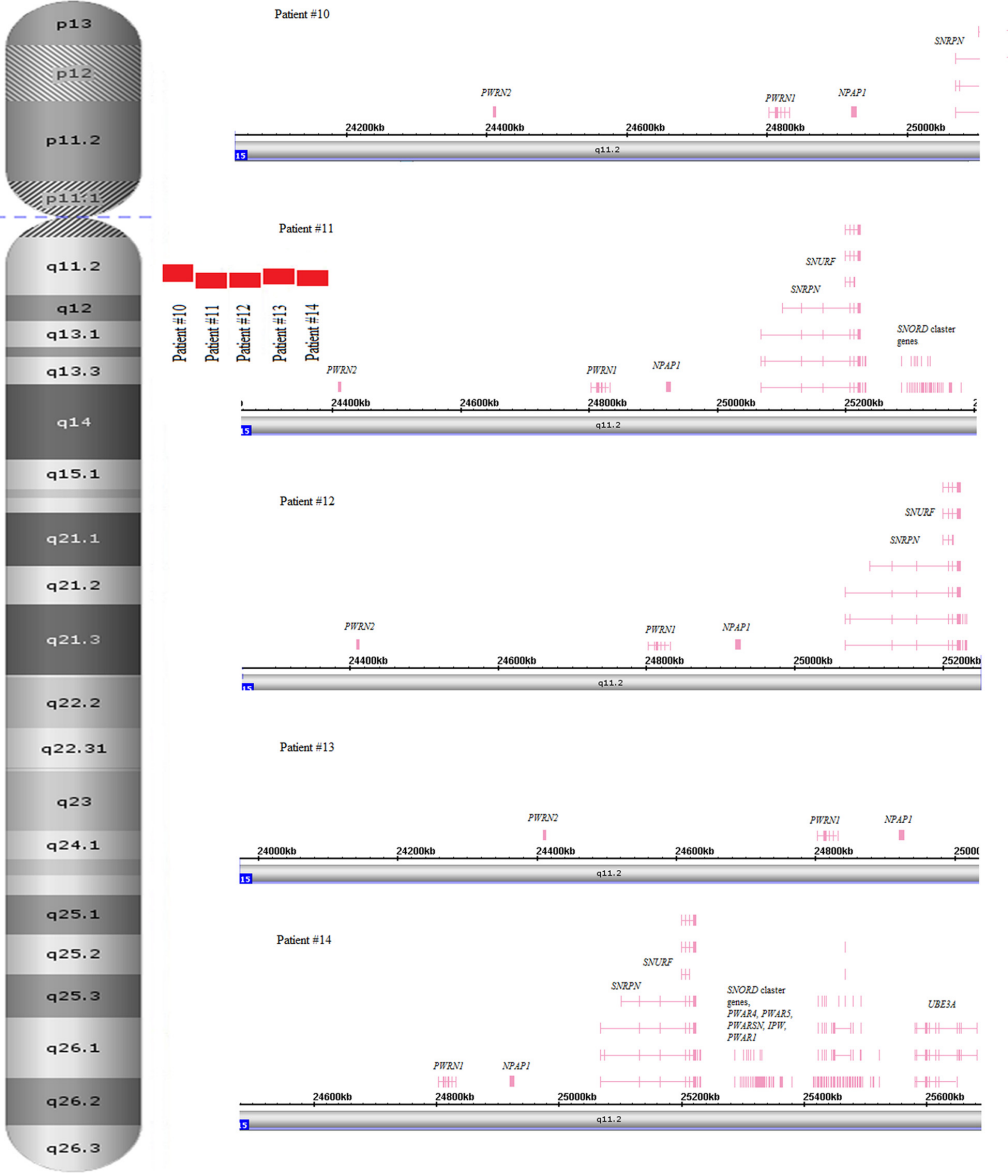
Schematic depiction of chromosomal and genomic regions affected by LCHS at 15q11.2 (5 patients) using Affymetrix Chromosome Analysis Suite software screenshots

LCSH at imprinted loci previously described as those of imprinting syndromes [34–36] were found in 14 cases (5.2 %). These were 2 cases of LCSH at 7q21.3 (SRS), 4 cases of LCSH at 7q31.2, 3 (SRS) (Fig. 1), LCSH at 11p15.5 (SRS/BWS) (Fig. 2), and LCSH at 15p11.2 (AS/PWS) (Fig. 3). Molecularly, LHCS spanned the loci containing imprinted genes checked by the GENEIMPRINT database (http://www.geneimprint.com/site/genes-by-species.Homo+sapiens). Apart from a case of LCSH at 7q31.33q32.3 (~4.3 Mb in size), the remaining LHCS spanned DNA sequences varied from 1 to 1.6 Mb. No correlation with the size of LCSH and disease’s phenotype was observed. The size of LCSH was indicative for excluding whole-chromosome uniparental disomy as the mechanism for phenotypic manifestations in these cases. Table 1 summarizes data on molecular, chromosomal and clinical features of LCSH at the imprinted chromosomal regions.

**Table 1:** Summary of LCSH, associated clinical findings, and imprinted genes

Case #	Chromosomal region	Age	Clinical features	Size, kb	Genes (imprinted)
1	7q21.3	5 years	Developmental delay, autistic behavior, hyperactivity	1098	*SGCE, PEG10 PPP1R9A*
2	7q21.3	11 years	Intellectual disability, developmental delay	1062	*SGCE, PEG10, PPP1R9A, TFPI2*
3	7q31.33q32.3	2 years	Intellectual disability, developmental delay, microcephaly, seizures, facial dysmorphisms, muscular hypotonia	4257	*KLF14, MEST, COPG2, MESTIT1, CPA4*
4	7q32.1q32.2	2 years 7 months	Intellectual disability, developmental delay, microcephaly, seizures, facial dysmorphisms	1089	*CPA4*
5	7q32.2	3 years	Intellectual disability, developmental delay, facial dysmorphisms	1033	*CPA4, MESTIT1, MEST, COPG2*
6	7q32.2	15 years	Intellectual disability, developmental delay, congenital heart defect	1020	*KLF14, MEST, COPG2, MESTIT1, CPA4*
7	11р15.5р15.4	5 years	Intellectual disability, autistic behavior, microcephaly, seizures, facial dysmorphisms, somatic overgrowth	1360	*CDKN1C, KCNQ1DN, KCNQ1, SLC22A18AS, SLC22A18, PHLDA2, NAP1L4, OSBPL5,*
8	11р15.5р15.4	10 years	Developmental delay, autistic behavior, facial dysmorphisms, somatic overgrowth	1147	*IGF2, H19, CDKN1C, KCNQ1DN, KCNQ1, SLC22A18, PHLDA2, NAP1L4, OSBPL5, IGF2AS, INS, TH, ASCL2, TSPAN32, CD81, TSSC4, TRPM5, KCNQ1OT1, SLC22A18AS*
9	11p15.5p15.4	4 years	Intellectual disability, developmental delay, macrocephaly, feeding difficulty, umbilical hernia, hepatomegaly, undescended testis, facial dysmorphisms, short neck	1554	*CDKN1C, KCNQ1, KCNQ1OT1, ASCL, TSPAN32, CD81, TSSC4, TRPM5, SLC22A18AS, SLC22A18, PHLDA2 NAP1L4, OSBPL5*
10	15q11.2	2 years 10 months	Intellectual disability, developmental delay, feeding difficulty, facial dysmorphisms	1068	*NPAP1, SNRPN*
11	15q11.2	4 years	Intellectual disability, developmental delay, hyperactivity, facial dysmorphisms, seizures	1158	*SNRPN, PAR1, IPW, PAR5,*
12	15q11.2	5 years	Developmental delay, autistic behavior, seizures, facial dysmorphisms	1002	*NPAP1, SNRPN*
13	15q11.2	5 years	Developmental delay, autistic behavior, fetal cerebral ventriculomegaly, facial dysmorphisms	1067	*NPAP1*
14	15q11.2	18 years	Intellectual disability, personality disorder	1224	*NPAP1, SNRPN, SNURF, SNORD107, SNORD108, SNORD109B, SNORD109A UBE3A*

## Discussion

Epigenomic variations and instability are known to be associated with human diseases [8–12, 21, 24, 30]. Here, a primary imprinting defect (according to previous classification of imprinting defects [37]) is described. Along with chromosomal abnormalities and CNV (germline and somatic) [1–7, 38–40], these epigenetic mutations can be considered as a common cause of neurodevelopmental diseases. There are no known epigenetic/epigenomic alterations detectable as common as LCSH at imprinted loci that can be considered as a causative for ID, autism or epilepsy. It is noteworthy, that LCSH are detectable by SNP chromosomal microarrays only [18, 20, 21, 32, 41, 42]. Although imprinting defects similar to LCSH can be also detected by molecular genetic approaches (methyl-sensitive polymerase chain reaction, bisulfite sequencing etc.) [36, 41, 43], these techniques are poorly effective for the detection because of their targeted nature. SNP chromosomal microarrays may lead to a 5 % improvement in etiological yield by uncovering LCSH at imprinted loci.

Clinically, 14 cases of LCSH spanning shortly the imprinted loci weakly resembled SRS, BWS, AS or PWS [10, 34–36, 43, 44]. Additional phenotypic features have been observed, as well (Table 1). In total, phenotypic manifestations in these cases have not allowed attributing them to a specific imprinting syndrome providing speculations about causative relationship between unexplained ID, autism and epilepsy and LCSH at imprinted loci.

Since LCSH were observed in all the individuals of the cohort, we have compared our results with previous studies of clinical and unaffected populations [13–23]. Identical LCSH in unrelated individuals were found to be confined to specific regions (i.e., 3p21 and 16p11.2p11.1). These were detected in the majority of patients. Consequently, we concluded that these LCSH are the result of a technological drawback. However, specific organization of these genomic loci can manifest as LCSH during SNP chromosomal microarray analysis. In the available literature, 7q21.3, 7q31.2, 11p15.5, and 15p11.2 genomic loci were not described as consistently affected by LCSH [13–20]. Although Wang et al. 2015 [21] have reported large LCSH to affect 11p and 15q, these occasional cases are likely to represent rare cases of uniparental disomy associated with corresponding imprinting disorders. Accordingly, these epigenomic mutations are unlikely to represent the same short LCSH reported in the present study.

Mechanisms and consequences of LCSH are poorly understood [13–15, 45]. Accordingly, the interpretation of these epigenetic mutations can represent a challenge. To solve the problem *in silico*, one can apply a variety of bioinformatic approaches to gene prioritization, which are known to be effective for uncovering functional significance of genomic and epigenomic variations [40, 46, 47]. Molecular testing for AS and PWS performed previously (fluorescence *in situ* hybridization-based and methylation analyses) [48] was not able to uncover these epigenetic mutations. Therefore, it is important to note that alternative empirical methods giving a solution to this problem do not currently exist.

## Conclusions

This molecular cytogenetic and bioinformatic study shows for the first time that LCSH of 1–1.6 Mb in size at imprinted chromosomal regions (7q21.3, 7q31.2; 11p15.5; and 15p11.2) are relatively frequent (~5 %) among the children with intellectual disability, autism, congenital malformations and/or epilepsy. Thus, these epigenetic mutations appear to be common in neurodevelopmental diseases. Hence, to increase the diagnostic yield of SNP chromosomal microarrays, an additional consideration of shorter LCSH is warranted in children with intellectual disability, autism, congenital malformations and/or epilepsy.

## Methods

### Patients

Cases (*n* = 267) included in this study are a part of the Russian cohort of children with intellectual disability, autism, epilepsy and congenital anomalies partially described previously [49–51]. Written informed consent was obtained from at least one of the patients’ parents.

### SNP chromosomal microarray

CNV and LCSH were analyzed by CytoScan HD Arrays (Affymetrix, Santa Clara, CA) consisting of about 2.7 million markers for CNV evaluation and about 750,000 SNPs for LCSH analysis. The laboratory procedures have been previously described in detail [17, 18, 21, 51, 52]. CNV and LCSH were visualized by the Affymetrix Chromosome Analysis Suite software (ChAS analysis files for CytoScan® HD Array version NA32.3). Genomic localization was defined using NCBI Build GRCh37/hg19 reference sequence. Imprinted genes were checked against the Geneimprint database (http://www.geneimprint.com).

### Bioinformatics

Bioinformatic analyses were performed using an original approach to gene and CNV prioritization as described in our earlier papers [46, 51, 53]. This procedure was performed to exclude the phenotypic effect of CNV and to confirm clinical relevance of LCSH. Briefly, the prioritization was performed using ontology-based gene filtering/ranking and fusion of data acquired from clinical, genomic, epigenetic, proteomic, and metabolomic databases as well as interactomic software.

## Abbreviations

AS: Angelman syndrome
BWS: Beckwith-Wiedemann syndrome
CNV: Copy number variations
HMZ: Homozygosity
ID: Intellectual disability
LCSH: Long contiguous stretches of homozygosity
PWS: Prader-Willi syndrome
SRS: Silver-Russell syndrome
SNP: Single-nucleotide polymorphism
